# Insulin‐like growth factor 1 promotes proliferation and invasion of papillary thyroid cancer through the STAT3 pathway

**DOI:** 10.1002/jcla.23531

**Published:** 2020-08-26

**Authors:** Li Yang, Zenghuan Tan, Yukun Li, Xueqiang Zhang, Yiping Wu, Baoyuan Xu, Mei Wang

**Affiliations:** ^1^ Department Ⅱ of Endocrinology Handan Central Hospital Handan China; ^2^ Department Ⅱ of Endocrinology Third Affiliated Hospital of Hebei Medical University Shijiazhuang China; ^3^ Department of Otolaryngology Handan Central Hospital Handan China; ^4^ Department of Neurology Handan Central Hospital Handan China; ^5^ Department of Pediatric Handan Central Hospital Handan China

**Keywords:** IGF1, invasion, papillary thyroid cancer, proliferation, STAT3

## Abstract

**Background:**

Papillary thyroid cancer (PTC) is a kind of thyroid cancer. Previous studies showed that insulin‐like growth factor‐1 (IGF1) plays an important role in tumorigenesis, development, invasion, and metastasis. However, the function of IGF1 in PTC progression remains unclear.

**Methods:**

Seventy‐three pairs of PTC tissue specimens and adjacent normal specimens form and normal cell line and PTC cell lines were collected in this study. The immunohistochemistry (IHC) assay was performed to test the expression of IGF1. The RNA isolation and quantitative real‐time PCR assays (qRT‐PCR assays) and Western blot analysis were used to test mRNA and protein expression. Cell proliferation assay, EdU assay, flow cytometry assay, wound healing assay, and Transwell invasion assay were performed to test cell proliferation, invasion, and apoptosis.

**Results:**

We found that the expression of IGF1 in PTC tissue samples was higher than that in adjacent normal specimens and was significantly associated with tumor size, TNM staging, and lymph node metastasis. Furthermore, IGF1 treatment significantly increased cell viability in a dose‐dependent manner. EdU assay also demonstrated the effect of IGF1 on the proliferation of BCPAP and TPC1 cells. Moreover, IGF1 treatment effectively increased the invasive capacity of BCPAP and TPC1 cells. More importantly, IGF1 treatment could significantly enhance the phosphorylation of STAT3 in BCPAP and TPC1 cells. Moreover, cryptotanshinone (Cryp) treatment reversed the effect of IGF1 treatment on cell viability and invasion of BCPAP and TPC1 cells.

**Conclusion:**

Collectively, IGF1 promotes proliferation and invasion of PTC progression through the STAT3 signaling pathway.

## Graphical abstract

IGF1 promotes proliferation and invasion of PTC through STAT3 pathway.

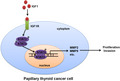

## 1 INTRODUCTION

Papillary thyroid cancer (PTC) is a kind of thyroid cancer, which is the most common endocrine malignancy. It has been reported that 85%‐90% of thyroid cancer patients belong to PTC.^1^ Although PTC patients at the early stage had a good response to thyroidectomy treatment, the recurrence and metastasis result in poor prognosis. Therefore, it is still of great significance to understand the molecular mechanism of PTC progression.

As a potent mitogen for many types of cells, insulin‐like growth factor‐1 (IGF1) binds with IGF‐1R and can regulate multiple biological functions, such as cell proliferation, differentiation, and apoptosis. Previous evidence suggested that IGF1 plays an important role in tumorigenesis, development, invasion, and metastasis. In melanoma, IGF1 inhibition prevented malignant cell proliferation, migration and invasion, and lung colony formation in immunodeficient mice.^2^ Suren et al showed that IGF1 upregulated Cyr61 primarily induced breast cancer cell proliferation and invasion through activation of the AKT‐PI3K pathway.^3^ IGF1/IGF1R/STAT3 signaling promoted IFITM2 expression and gastric cancer growth and metastasis.^4^ Moreover, the expression of IGF1 and IGF1R was significantly higher in follicular adenomas nodular goiters and PTC than that in the controls.^5^ However, the function of IGF1 in PTC progression remains unclear.

In this study, we examined the IGF1 expression level in clinical PTC tissue specimens and found that the expression of IGF1 in PTC tissue samples was higher than that in adjacent normal specimens and was significantly associated with tumor size, TNM staging, and Lymph node metastasis. Furthermore, IGF1 treatment significantly increased cell viability and proliferation of BCPAP and TPC1 cells. Moreover, IGF1 treatment effectively increased the invasive capacity of BCPAP and TPC1 cells. More importantly, Cryp treatment reversed the effect of IGF1 treatment on cell viability and invasion of BCPAP and TPC1 cells. Therefore, the data in our study suggest potential roles of IGF1 in the regulation of cell growth and invasion in PTC.

## 2 MATERIALS AND METHODS

### 2.1 Subjects

We collected 73 pairs of PTC tissue specimens and adjacent normal specimens form PTC patients in Handan Central Hospital. The details of PTC patients are shown in Table 1. The study protocol was approved by the Institutional Ethics Committee of Handan Central Hospital. All patients provided written informed consent.

**Table 1:** Association between IGF1 expression and clinicopathological features

Clinicopathological features	N	IGF1 expression		*P* Value
		Low	High	
Age (y)				
≤58	36	16	20	.362
>58	37	15	22	
Gender				
Female	30	14	16	.457
Male	43	18	25	
Tumor size (cm)				
≤2	28	22	6	.039*
>2	45	11	34	
TNM stage				
I + II	30	18	12	.047*
III + IV	33	9	24	
Lymph node metastasis				
Positive	46	13	33	.041*
Negative	27	10	17	

Abbreviation: TNM, tumor node metastasis.

* Statistically significant (*P* < .05).

### 2.2 Immunohistochemistry

The immunohistochemistry (IHC) assay was performed as previously described.^6^ Briefly, after deparaffinized, rehydrated, and antigen retrieval, the sections were incubated with rabbit anti‐IGF1 (Abcam) overnight at 4°C. The sections were incubated with a biotinylated secondary antibody and stained with hematoxylin. Following this, the slides were imaged using the ScanScope microscope (Olympus).

The IGF1 expression based on IHC assay was assessed as previously described^7^ and examined by two pathologists, who were blinded to the identity of specimens.

### 2.3 Cell culture

Normal cell line Nthy‐ori3‐1 and PTC cell lines K1, BCPAP, and TPC1 were obtained from Shanghai Huiying Biological Technology Co., Ltd. All cell lines were cultured in RPMI‐1640 medium supplemented with 10% fetal bovine serum (FBS) at 37°C in a humidified atmosphere containing 5% CO_2_.

### 2.4 RNA isolation and quantitative real‐time PCR assays (qRT‐PCR assays)

Total RNA was extracted from cells using TRIzol reagent (Invitrogen) according to the manufacturer's instructions. Takara PrimeScript RT reagent (Takara Bio, Inc) was used to synthesize the first‐strand cDNA. Following this, we used the SYBR Green PCR reagent kit (Takara Bio, Inc) to perform qRT‐PCR assay following the manufacturer's instructions. β‐actin was used as an internal control. The qRT‐PCR primer sequences in this study were used as IGF1 Forward Primer 5′‐GCTCTTCAGTTCGTGTGTGGA‐3′, Reverse Primer 5′‐GCCTCCTTAGATCACAGCTCC‐3′; MMP‐2 Forward Primer 5′‐TGACTTTCTTGGATCGGGTCG‐3′, Reverse Primer 5′‐AAGCACCACATCAGATGACTG‐3′; MMP‐9 Forward Primer 5′‐TGTACCGCTATGGTTACACTCG‐3′, Reverse Primer GGCAGGGACAGTTGCTTCT; β‐Actin Forward Primer 5′‐GTTGAGAACCGTGTACCATGT‐3′, Reverse Primer 5′‐TTCCCACAATTTGGCAAGAGC‐3′.

### 2.5 Western blot

Total protein was extracted from the indicated cells with RIPA buffer containing protease inhibitors (Beyotime). The Western blot analysis was performed as previously described^8^ The first antibodies in this study included rabbit anti‐IGF1 (Abcam), mouse anti‐MMP2 (Abcam), mouse anti‐MMP9 (Abcam), rabbit anti‐STAT3 (CST), rabbit anti‐p‐STAT3 (CST), and mouse anti‐GAPDH (Abcam). The band density was analyzed using ImageJ (NIH).

### 2.6 Cell proliferation assay

BCPAP and TPC1 cells were seeded in 96‐well plates, treated with IGF1 at a different dose (0, 2.5 ng, 5 ng, 10 ng, and 20 ng), and cultured at 37°C for 48 hours. We added 10 µL Cell Counting Kit‐8 (Beyotime) into the cells. The absorbance was measured at 450 nm.

### 2.7 EdU assay

EdU cell proliferation (Ribo) was performed as previously described.^9^ Briefly, BCPAP and TPC1 cells treated with EdU for 2 hours. Following this, the cells were stained with DAPI and imaged using the ScanScope microscope.

### 2.8 Flow cytometry

For cell cycle distribution, BCPAP and TPC1 cells were plated in 6‐well plates and treated with IGF1 (10 ng) for 24 hours. The cells were fixed in 70% ethanol containing 0.5% FBS at −20°C for 24 hours. Then, the cells were treated with cell cycle assays (Fcmrcs, Nanjing, Jiangsu, China) and analyzed by flow cytometry.

### 2.9 Wound healing assay

BCPAP and TPC1 cells were plated in 12‐well plates. When the cells reached 90%‐100% of confluence, the cell monolayer was wounded using a sterile pipette tip and treated with IGF1 (10 ng) for 24 hours. The cells were photoimaged longitudinally, and the relative wound healing rate was measured 0 and 24 hours post‐wounding.

### 2.10 Transwell invasion assay

For invasion assay, 8 μm pore size transwell chambers (Corning Incorporated) was pre‐coated with Matrigel matrix (BD Biosciences). Briefly, BCPAP and TPC1 cells in serum‐free medium were seeded into the upper chamber. The lower chamber was filled with complete medium. After 48 hours, cells in the down chamber were fixed and counted using a light microscope.

### 2.11 Statistical analysis

Student's *t* test was used to measure statistical significance. The data were displayed as mean ± standard deviation (SD). *P* < .05 was considered as statistical significance.

## 3 RESULTS

### 3.1 IGF1 was upregulated in PTC tissue specimens and cell lines

**FIGURE 1:**
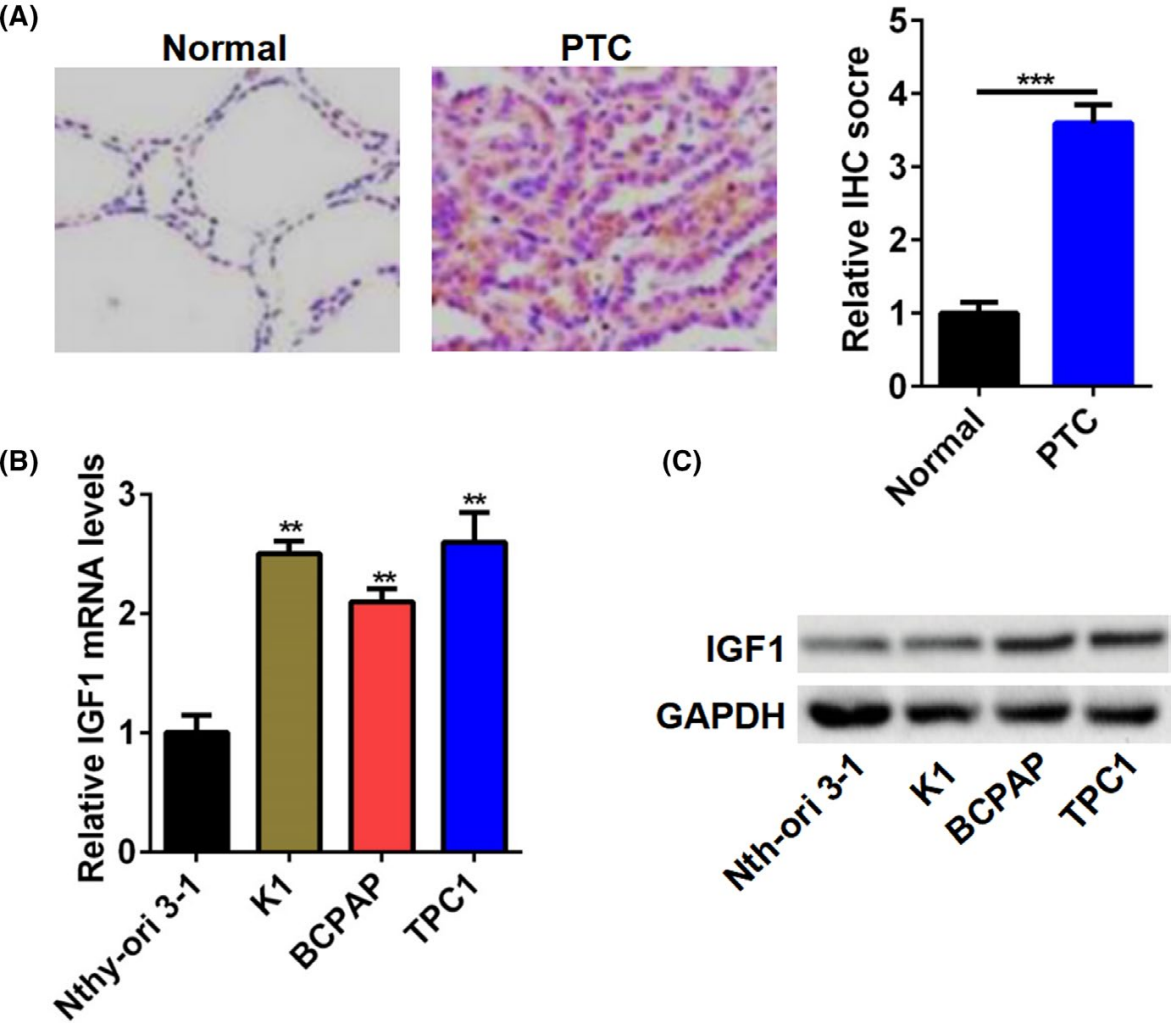
IGF1 was upregulated in PTC tissue specimens and cell lines. A, Representative image of IHC staining for IGF1 in PTC tissues. Quantitation of IGF1‐positive tumor cells based on IHC staining. B and C, The IGF1 mRNA (B) and protein (C) expression in PTC cell lines (K1, BCPAP, and TPC1 cell) and Nthy‐ori3‐1 cells. **P < .01, ***P < .001

Firstly, to investigate the relationship between IGF1 and PTC, we analyzed the expression of IGF1 in PTC tissue samples. The expression of IGF1 in PTC tissue samples was higher than that in adjacent normal specimens (Figure 1A and Figure S1). Similarly, both mRNA and protein levels of IGF1 were also higher in PTC cell lines, including K1, BCPAP, and TPC1 cells than that in Nthy‐ori3‐1 cells (Figure 1B,C). Additionally, we analyzed the relationship between the IGF1 expression and clinicopathologic features of patients with PTC. As shown in Table 1, the levels of IGF1 were significantly associated with tumor size, TNM staging, and Lymph node metastasis. However, no significant associations were found between IGF1 expression and age and gender.

### 3.2 IGF1 treatment promoted BCPAP and TPC1 cell proliferation

**FIGURE 2:**
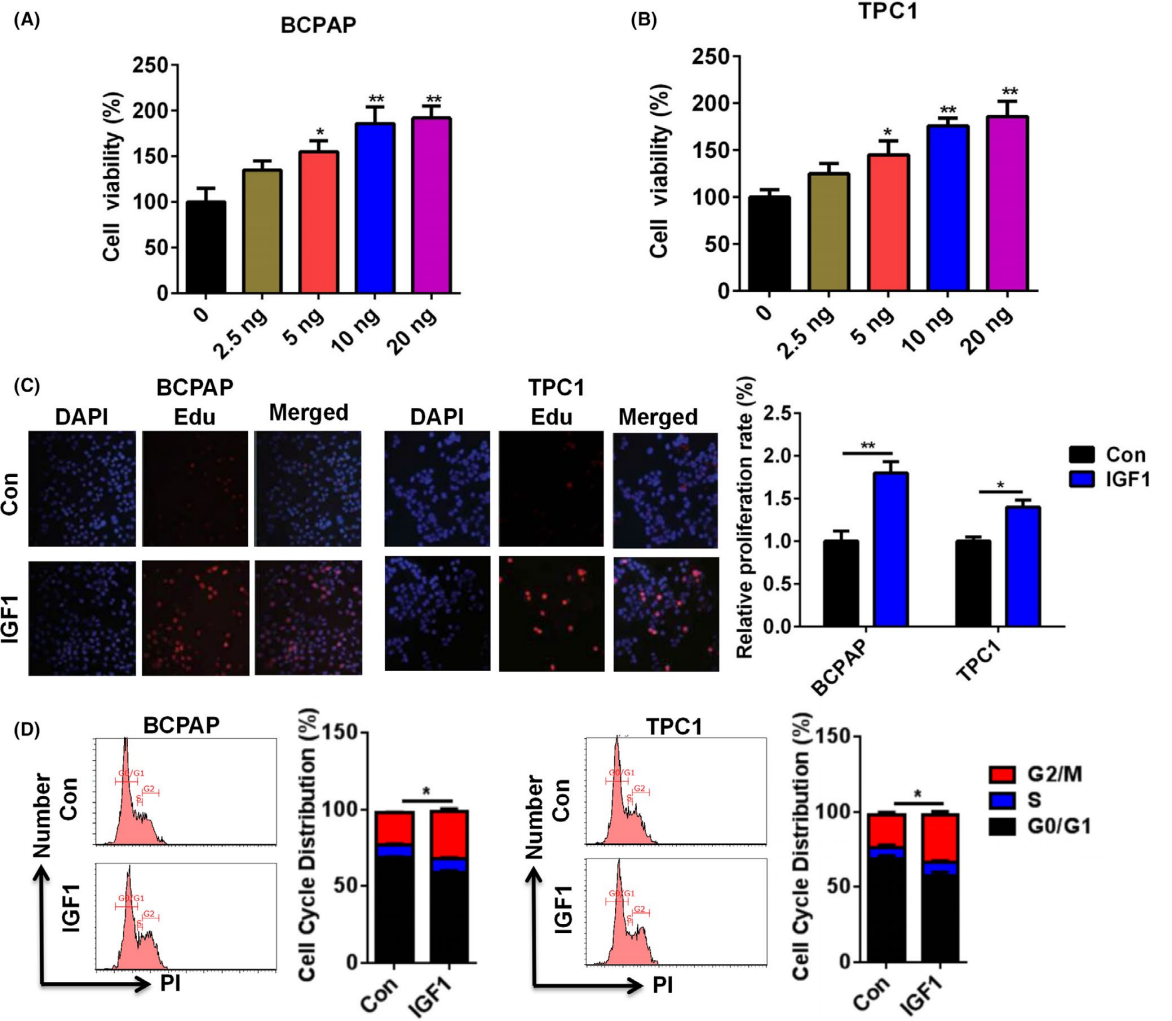
IGF1 treatment promoted the proliferation of BCPAP and TPC1 cells. A and B, Cell viability of BCPAP (A) and TPC1 (B) cells after treatment with IGF1 at a different dose (0, 2.5 ng, 5 ng, 10 ng, and 20 ng) was analyzed by CCK‐8. C, Cell proliferation of BCPAP and TPC1 cells after treatment with IGF1 (10 ng) was analyzed by EDU assay. D, Cell cycle distribution of BCPAP and TPC1 cells after treatment with IGF1 (10 ng) was analyzed by flow cytometry.*P < .05, **P < .01

BCPAP and TPC1 cells were selected to test the function of IGF1. BCPAP and TPC1 cells treated with IGF1 at a different dose (0, 2.5 ng, 5 ng, 10 ng, and 20 ng). As shown in Figure 2A,B, cell viability was significantly increased after IGF1 treatment, compared with the control group, and this promotion was dose‐dependent and 10 ng IGF1 exhibited the best‐promoting effect. Therefore, the IGF1 concentration of 10 ng was chosen for subsequent experiments. To confirm the data from cell viability, the EdU assay was performed. IGF1 treatment significantly enhanced the proliferation of BCPAP and TPC1 cells (Figure 2C). Moreover, cell cycle analysis demonstrated that IGF1 treatment increased the number of BCPAP and TPC1 cells in the G2/M and S phase (Figure 2C).

### 3.3 IGF1 treatment promoted BCPAP and TPC1 cell invasion

**FIGURE 3:**
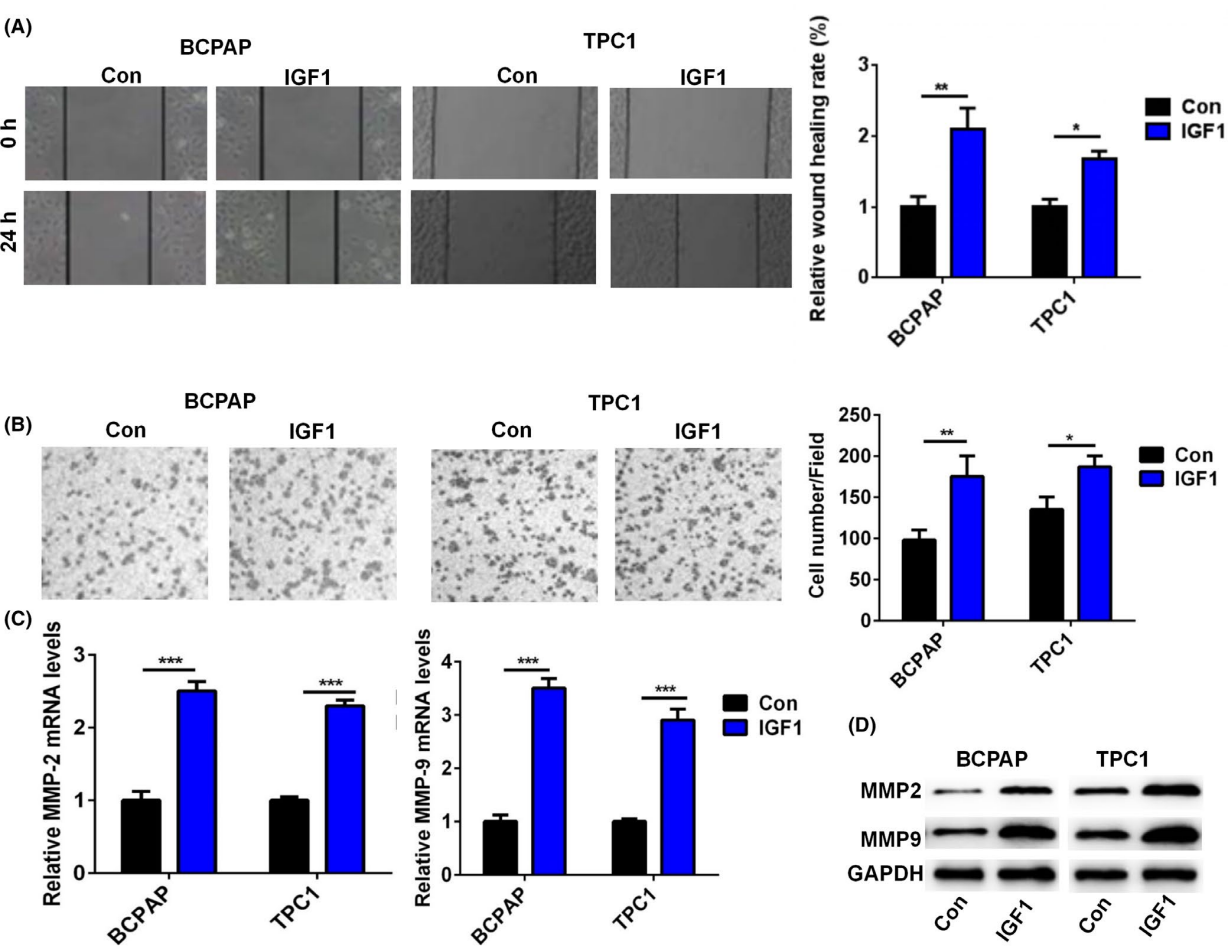
IGF1 treatment promoted cell invasion of BCPAP and TPC1 cells. A, The wound healing rate of BCPAP and TPC1 cells after treatment with IGF1 (10 ng) was analyzed. One representative image from three separate experiments is shown (original magnification 40×). B, The cell invasive capacity of BCPAP and TPC1 cells after treatment with IGF1 (10 ng) was analyzed by transwell invasion assay. One representative image from three separate experiments is shown (original magnification 40×). C, The relative mRNA level of MMP‐2 and MMP‐9 in BCPAP and TPC1 cells after treatment with IGF1 (10 ng) was analyzed by qRT‐PCR. D, The protein level of MMP‐2 and MMP‐9 in BCPAP and TPC1 cells after treatment with IGF1 (10 ng) was analyzed by Western blot.*P < .05, **P < .01, ***P < .001

Next, we determined the effect of IGF1 on the invasive capacities of BCPAP and TPC1 cells through wound healing and transwell invasion assays. As shown in Figure 3A, IGF1 treatment effectively increased the wound healing rate of BCPAP and TPC1 cells. In addition, IGF1 treatment enhanced the invasive capacity of BCPAP and TPC1 cells (Figure 3B). Moreover, IGF1 treatment significantly upregulated the mRNA and protein levels of MMP‐2 and MMP‐9 (Figure 3C,D). These results suggested that IGF1 associated with PTC invasion.

### 3.4 IGF1 activated the STAT3 pathway in BCPAP and TPC1 cells

**FIGURE 4:**
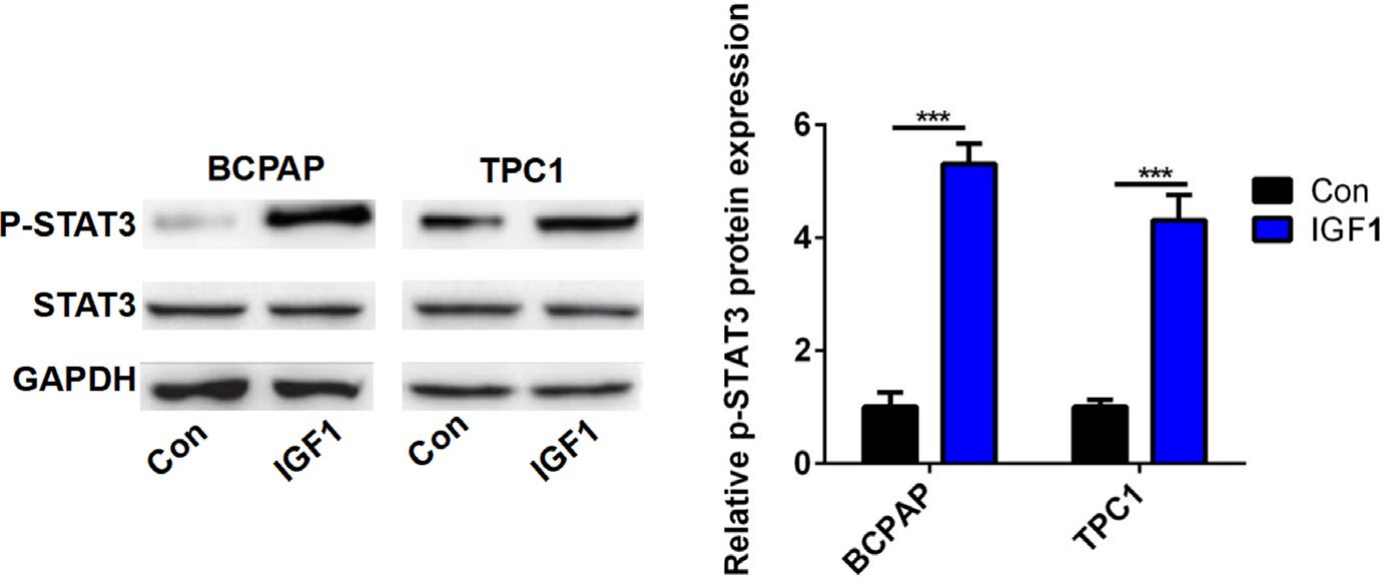
IGF1 activated the STAT3 pathway. The protein level of p‐STAT3 and STAT3 in BCPAP and TPC1 cells after treatment with IGF1 (10 ng) was analyzed by Western blot. The band density was analyzed using ImageJ. ***P < .001

To investigate the mechanisms underlying tumorigenesis of IGF1 in PTC cell lines, we examined the effect of IGF1 on the activities of the STAT3 signaling pathway. The Western blot data showed that IGF1 treatment could significantly enhance the phosphorylation of STAT3 in BCPAP and TPC1 cells (Figure 4). Therefore, IGF1 promoted PTC cell proliferation and invasion via activating the STAT3 signaling pathway.

### 3.5 The IGF1/STAT3 axis mediated the effect of IGF1 on cell growth in PTC

**FIGURE 5:**
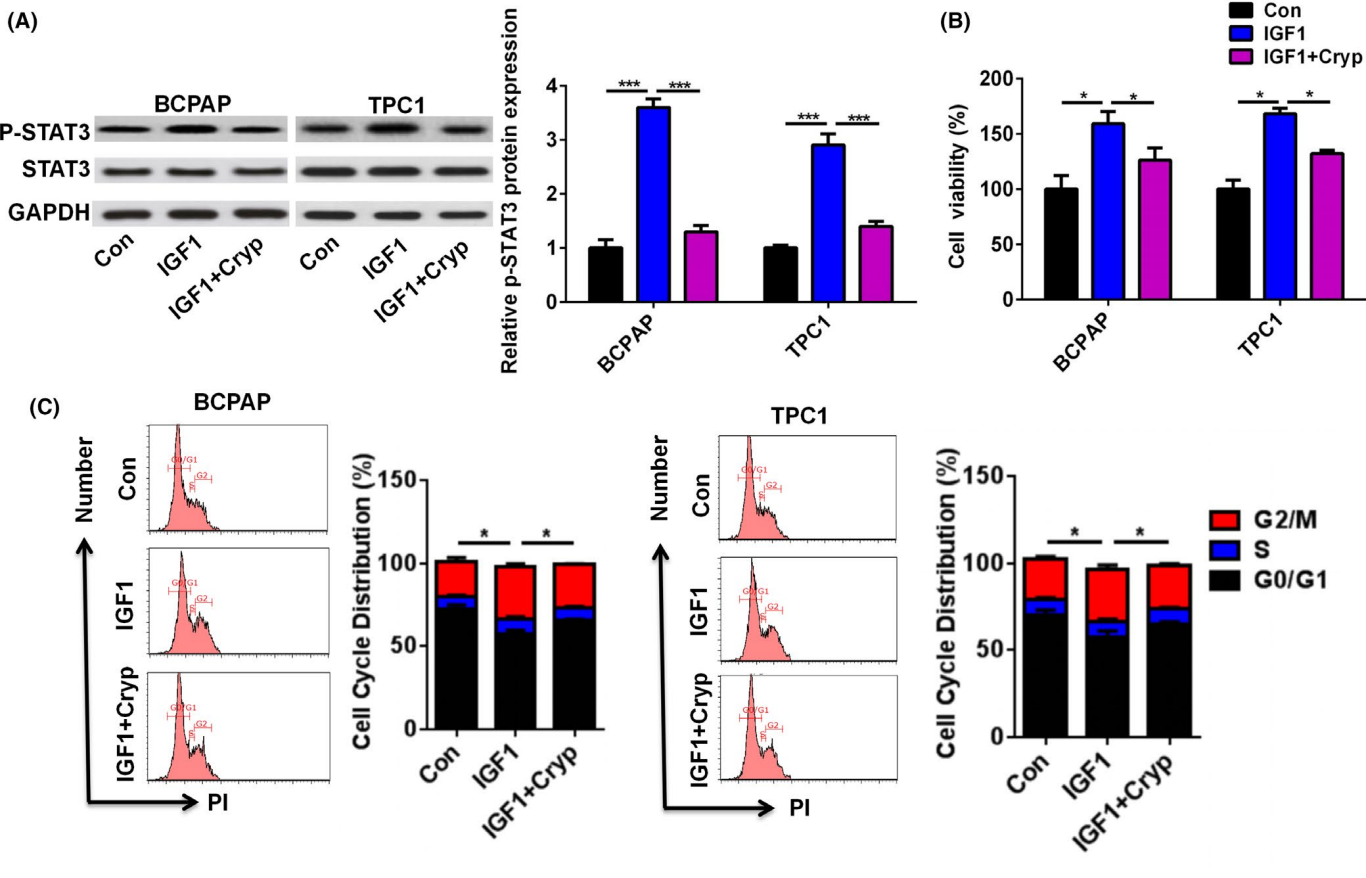
STAT3 axis inhibition reversed the effect of IGF1 treatment on cell viability of PTC cells (A) The protein level of p‐STAT3 and STAT3 in BCPAP and TPC1 cells after cotreatment with IGF1 (10 ng) and cryptotanshinone (Cryp, 10 µmol/L) was analyzed by Western blot. (B) Cell viability of BCPAP and TPC1 cells after cotreatment with IGF1 (10 ng) and Cryp was analyzed by CCK‐8. (C) Cell cycle distribution of BCPAP and TPC1 cells after cotreatment with IGF1 (10 ng) and Cryp was analyzed by flow cytometry. *P < .05, ***P < .001

In order to investigate whether the effect of IGF1 on cell growth was mediated by the STAT3 pathway, we cotreated BCPAP and TPC1 cells with STAT3 inhibitor cryptotanshinone (Cryp) and IGF1. As shown in Figure 5A, IGF1 treatment significantly increased the phosphorylation levels of STAT3. However, Cryp treatment abolished the effect of IGF1 treatment on the phosphorylation levels of STAT3 (Figure 5A). Moreover, Cryp treatment reversed the effect of IGF1 treatment on cell viability of BCPAP and TPC1 cells (Figure 5B). In addition, Cryp treatment abolished the effect of IGF1 treatment on the cell cycle distribution of BCPAP and TPC1 cells (Figure 5C).

### 3.6 The IGF1/STAT3 axis mediated the effect of IGF1 on cell invasion in PTC

**FIGURE 6:**
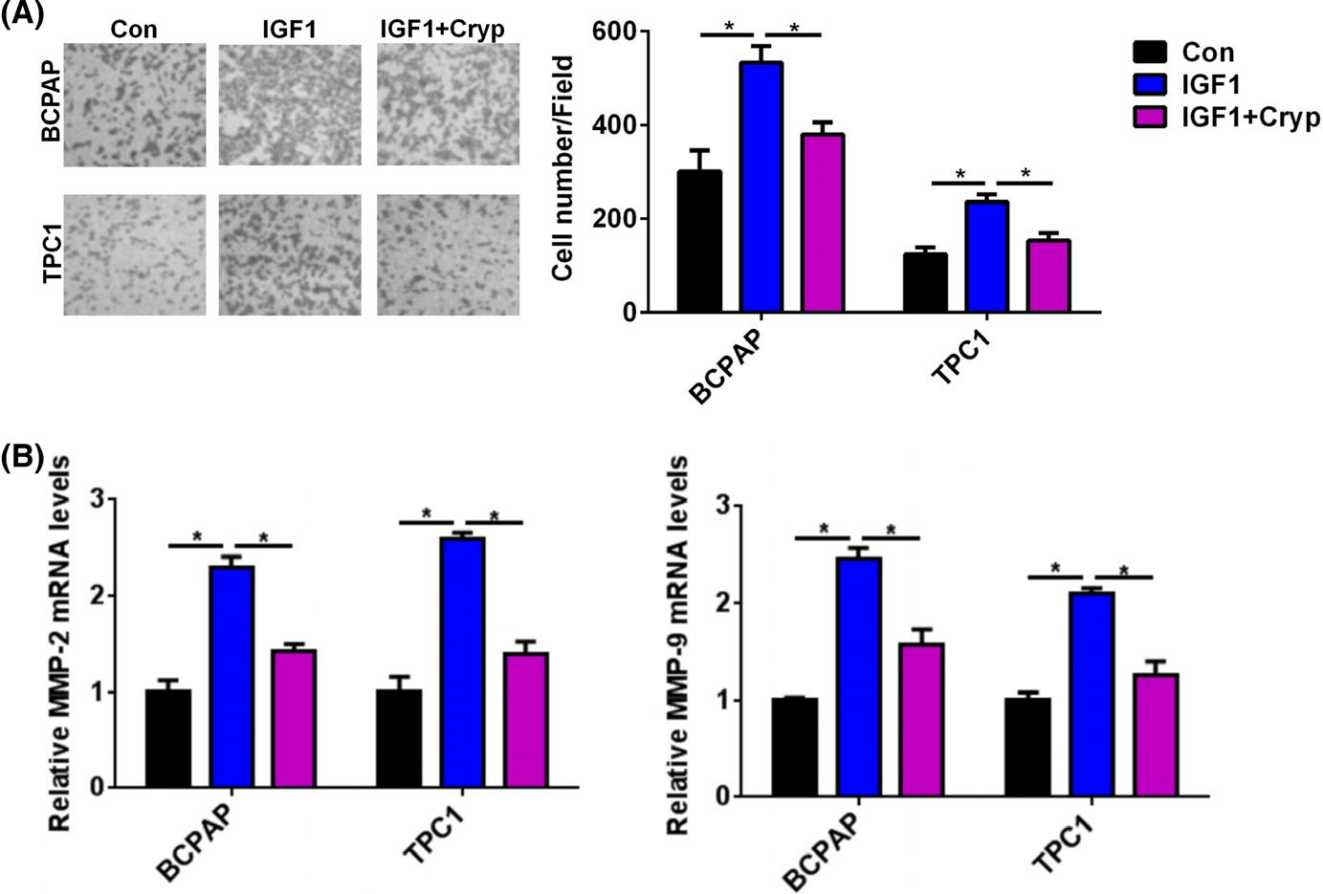
STAT3 axis inhibition reversed the effect of IGF1 treatment on cell invasion of PTC cells. A, The cell invasive capacity of BCPAP and TPC1 cells after cotreatment with IGF1 (10 ng) and Cryp was analyzed by transwell invasion assay. B, The relative mRNA level of MMP‐2 and MMP‐9 in BCPAP and TPC1 cells after cotreatment with IGF1 (10 ng) and Cryp (10 µmol/L) was analyzed by qRT‐PCR *P < .05

We next demonstrated the effect of the IGF1/STAT3 axis on cell invasion in PTC. As shown in Figure 6A, IGF1 treatment effectively increased the invasive capacity of BCPAP and TPC1 cells, while Cryp treatment abrogated the promotion of IGF1 treatment on cell invasion in the BCPAP and TPC1 cells. In addition, Cryp treatment reversed the promotion of IGF1 treatment on the mRNA expression of MMP2 and MMP‐9 in the BCPAP and TPC1 cells (Figure 6B). These results suggested that the IGF1/STAT3 axis mediated the effect of IGF1 on cell invasion in PTC.

## 4 DISCUSSION

Growing evidence indicates that IGF1/IGF1R is found highly expressing in multiple types of cancer such as colorectal cancer, non‐small cell lung cancer, prostate cancer, and gastrointestinal stromal tumors.^10^, ^11^, ^12^, ^13^ Moreover, IGF‐1 and IGF‐1R protein and mRNA expression were significantly higher in follicular adenomas, nodular goiters, and PTC than those in the controls.^5^ Also, the serum IGF1 levels were associated with the histologic characteristics of thyroid cancer.^14^ In our study, we performed the IHC assay to detect the expression of IGF1 in PTC tissue samples and found that the expression of IGF1 in PTC tissue samples was higher than that in adjacent normal specimens. Furthermore, the levels of IGF1 were significantly associated with tumor size, TNM staging, and Lymph node metastasis. More importantly, both mRNA and protein levels of IGF1 were also higher in PTC cell lines, including K1, BCPAP, and TPC1 cells than that in Nthy‐ori3‐1 cells. Hence, high expression of IGF1 may be an important factor in regulating the progression of PTC.

IGF1 has been reported to regulate tumor progression in multiple cancers. For example, IGF1 could increase cell migration, invasion, and survival of colorectal cancer cells via the AKT pathway.^15^ IGF‐1 inhibition prevented malignant cell proliferation, migration and invasion, and lung colony formation in immunodeficient mice through an epithelial‐mesenchymal transition process in melanoma.^2^ IGF1 was able to activate the AKT‐PI3K and MAPK pathways and upregulate Cyr61, which induced breast cancer growth and invasion.^3^ In epithelial ovarian cancer, the IGF1/AKT pathway was involved in vitamin D‐binding protein‐mediated cell aggressiveness.^16^ Barbara et al reported that IGF1 regulated PKM2 function and glycolysis through AKT phosphorylation in cancer cells.^17^ In gastric cancer, IGF1/IGF1R/STAT3 signaling‐inducible IFITM2 promotes gastric cancer growth and metastasis.^4^ In glioblastoma multiforme, IGF1/IGF1R signaling promoted survival and suppressed apoptosis of glioblastoma cells through the PI3K/AKT pathway.^18^ A similar observation was found in our study. IGF1 treatment significantly increased cell viability in a dose‐dependent manner. EdU assay also demonstrated the effect of IGF1 on the proliferation of BCPAP and TPC1 cells. Cell cycle analysis showed that IGF1 treatment increased the number of BCPAP and TPC1 cells in the G2/M and S phase. Moreover, IGF1 treatment effectively increased the invasive capacity of BCPAP and TPC1 cells and upregulated the levels of MMP‐2 and MMP‐9 in PTC cells. Thus, IGF1 played important roles in the regulation of cell growth and invasion in PTC.

Previous studies showed that the biological role of STAT3 pathway in PTC progression is controversial. Joana et al showed that activated STAT3 negatively regulated thyroid tumorigenesis.^19^ By contrast, IL6/STAT3 axis mediated resistance to BRAF inhibitors in thyroid carcinoma cells.^20^ SIX1, a tumor promoter, promoted the proliferation and invasion of thyroid carcinoma via activation of STAT3 signaling and its downstream targets in an EYA1‐dependent manner.^21^ Khan et al reported that curcumin synergistically enhanced the anticancer activity of cisplatin in PTC cells and in cancer stem‐like cells by targeting STAT3.^22^ Calcitriol treatment enhanced doxorubicin‐induced apoptosis in PTC cells through the regulation of the VDR/PTPN2/p‐STAT3 signaling pathway.^23^ Furthermore, STAT3 signaling was reported to be involved in IGF1/IGF1R‐mediated cell growth and metastasis in gastric cancer.^4^ Herein, we hypothesized that IGF1 regulated cell growth and invasion in PTC via the STAT3 signaling pathway. IGF1 treatment could significantly enhance the phosphorylation of STAT3 in BCPAP and TPC1 cells. Moreover, Cryp treatment reversed the effect of IGF1 treatment on cell proliferation and invasion of BCPAP and TPC1 cells. These results suggested that the IGF1/STAT3 axis mediated the effect of IGF1 on cell growth and invasion in PTC.

## 5 CONCLUSION

In summary, our study demonstrated that IGF1 was overexpressed in PTC tissue and was significantly associated with tumor size, TNM staging, and Lymph node metastasis. IGF1 treatment significantly increased the cell viability and invasive capacity of BCPAP and TPC1 cells via the STAT3 signaling.

## AUTHOR CONTRIBUTIONS

Li Yang designed experiments; Zenghuan Tan and Yukun Li carried out experiments; Xueqiang Zhang analyzed experimental results and analyzed sequencing data; YipingWu developed analysis tools; Baoyuan Xu and Mei Wang assisted with Illumina sequencing; Li Yang wrote the manuscript.

## Supporting information

Fig S1
