# Supplementary material for: Insulin‐like growth factor 1 promotes proliferation and invasion of papillary thyroid cancer through the STAT3 pathway
Source: J Clin Lab Anal. 2020 Aug 26;34(12):e23531. doi: 10.1002/jcla.23531 (PMC7755808; doi:10.1002/jcla.23531)
Supplement: Supplementary file 1 — Fig S1 [file JCLA-34-e23531-s001.doc]

**Supplemental Figure 1**


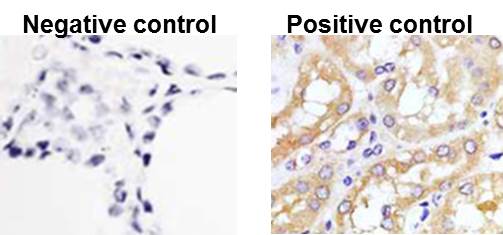


Supplemental Figure 1. The negative and positive control of IHC assays for IGF1 expression in PTC tissue specimens.
